# Mirizzi’s syndrome masquerading as cholangiocarcinoma: a case report

**DOI:** 10.1186/1752-1947-6-157

**Published:** 2012-06-15

**Authors:** Muhammad Rizwan Khan, Sameer ur Rehman

**Affiliations:** 1 Department of Surgery and Medical College, Aga Khan University & Hospital, Stadium Road, Karachi, 74800, Pakistan; 2 Department of Surgery, Aga Khan University & Hospital, Stadium Road, Karachi, 74800, Pakistan

**Keywords:** Mirizzi’s syndrome, Cholangiocarcinoma, Obstructive jaundice

## Abstract

**Introduction:**

Mirizzi’s syndrome is a rarely observed disorder that presents with obstructive jaundice. The condition is caused by a stone impacted in the gall bladder neck or cystic duct that impinges on the common hepatic duct, with or without a cholecystocholedochal fistula. The condition is often confused with other serious conditions such as hilar cholangiocarcinoma, which present with similar clinical and imaging findings, and a pre-operative diagnosis may be a serious challenge.

**Case presentation:**

We present the case of a 44-year-old Asian man with Mirizzi’s syndrome who was initially diagnosed as having cholangiocarcinoma based on his clinical presentation, raised cancer antigen 19–9 levels and radiological findings. Our patient was diagnosed as having Mirizzi’s syndrome intra-operatively and subsequently a cholecystectomy was performed with restoration of biliary drainage. Careful clinical assessment during surgery with the help of intra-operative frozen section helped in establishing the definitive diagnosis and altered the surgical procedure for our patient.

**Conclusions:**

Pre-operative diagnosis of Mirizzi’s syndrome could be challenging as the clinical, biochemical and radiological presentation is similar to other conditions causing obstructive jaundice such as choledocholithiasis, bile duct stricture or cholangiocarcinoma. A high index of suspicion and careful surgical assessment may help in establishing a diagnosis and alter the clinical course for our patient.

## Introduction

Mirizzi’s syndrome (MS) is a rarely observed condition characterized by impaction of stones in the neck of the gall bladder or cystic duct, causing mechanical obstruction of the common hepatic duct and presents clinically as intermittent or persistent jaundice. This syndrome was first described in 1948 by an Argentinean surgeon, Pablo Mirizzi [1]. The accurate diagnosis of Mirizzi’s syndrome is of particular importance to surgeons as the condition may be confused with choledocholithiasis, bile duct stricture or cholangiocarcinoma on initial presentation and hence the surgical treatment is associated with a significantly increased risk of inadvertent bile duct injury [2,3]. We report the case of a 44-year-old man with Mirizzi’s syndrome who was initially diagnosed as having cholangiocarcinoma based on his clinical presentation, raised cancer antigen (CA)19-9 levels and radiological findings.

## Case presentation

**Figure 1:**
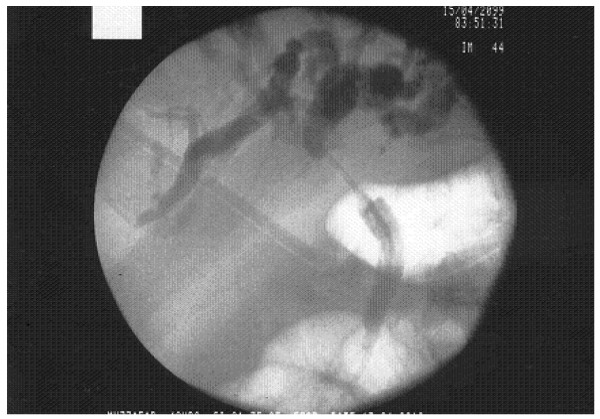
Initial cholangiogram during endoscopic retrograde cholangiopacreaticography showing a tight stricture of proximal common bile duct; a stent was placed in for biliary drainage.

A 44-year-old Asian man, an agriculturalist by occupation, presented to a local hospital with a four-day history of upper abdominal pain and vomiting. He also had history of progressively increasing jaundice for the last two months. He had significant comorbid conditions including hypertension and ischemic heart disease for which he underwent a coronary artery bypass grafting two years previously. A clinical diagnosis of cholangitis was made at presentation. He was resuscitated and an abdominal ultrasound (US) followed by endoscopic retrograde cholangiopacreaticography (ERCP) was performed. ERCP revealed a tight stricture involving the proximal bile duct extending to the confluence of right and left hepatic ducts (Figure 1). A plastic stent was placed to drain the biliary system. Unfortunately, the clinical course was complicated by ERCP-induced pancreatitis and our patient was transferred to our hospital for further management. On examination, he was deeply icteric and dehydrated, with marked tenderness in the upper abdomen. His blood investigations showed leukocytosis and markedly deranged liver functions with a total bilirubin level of 25.7mg/dL (normal range 0.1 to 1.2mg/dL), serum glutamic pyruvic transaminase of 27IU/L (normal range 0 to 45IU/L), serum glutamic oxaloacetic transaminase of 53IU/L (normal range 0 to 35IU/L), and alkaline phosphatase of 248IU/L (normal range 45 to 129IU/L). His serum amylase level was 118IU/L (normal range 28 to 100IU/L) and lactate dehydrogenase level was 790IU/L (normal range 266 to 500IU/L). Repeat US of the abdomen showed a distended gall bladder with multiple calculi within its lumen, moderate intra-hepatic biliary dilatation and no pneumobilia. He was resuscitated for two days, but his abdominal pain persisted and bilirubin level continued to rise. Subsequently, his biliary system was drained by a percutaneously placed external biliary drain and the previously placed stent was removed. The clinical condition of our patient gradually improved and he was started on an oral diet.

**Figure 2:**
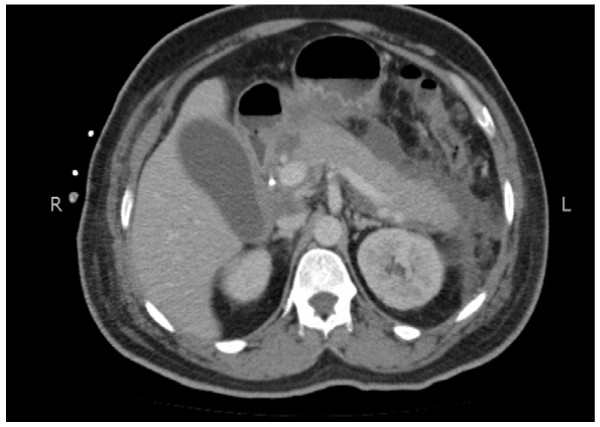
Axial view of the computed tomography scan of our patient showing soft tissue thickening at the gall bladder neck and proximal bile duct region.

**Figure 3:**
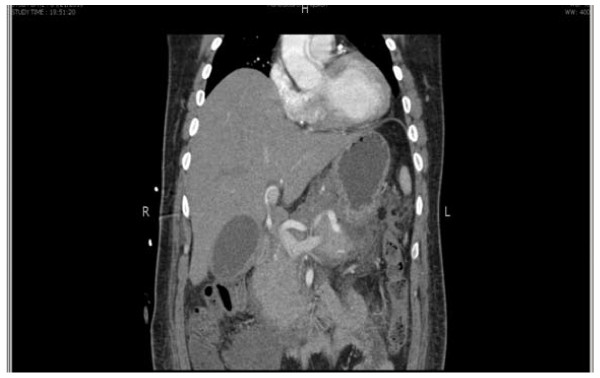
Sagittal view of the computed tomography (CT) scan of our patient showing soft tissue thickening at the gall bladder neck and proximal bile duct region.

In order to delineate the underlying pathology, further investigation was performed. His CA19-9 levels were found to be elevated at 71.10U/mL (normal range 0 to 33U/mL, median 5.0U/mL). A computed tomography (CT) scan of his abdomen showed moderately dilated intra-hepatic ducts up to the porta hepatis, soft tissue thickening involving the gall bladder neck and proximal bile duct and a few enlarged upper abdominal nodes. The findings were suggestive of neoplastic lesion involving the gall bladder neck and proximal common bile duct causing bile duct stricture (Figures 2 and 3). Taking into consideration the findings from the ERCP, CA19-9 level, and CT scan findings, a provisional diagnosis of cholangiocarcinoma involving the gall bladder neck was made.

**Figure 4:**
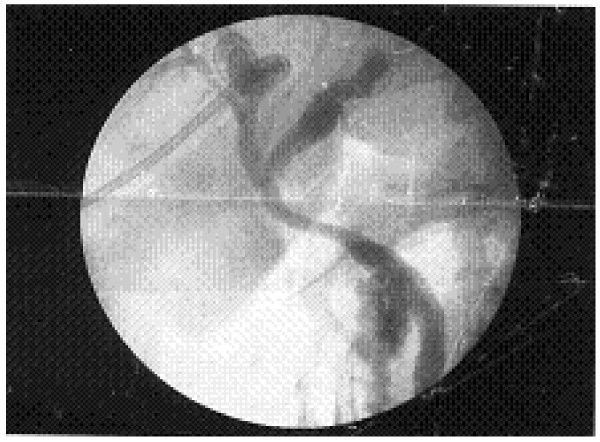
Intra-operative cholangiogram showing some narrowing of the bile duct due to external compression; note there is free flow of contrast into the duodenum.

Six weeks after his initial presentation, our patient was scheduled for resection of the tumor. On surgical exploration, a thick-walled gall bladder was seen with a large stone impacted in the cystic duct causing external compression on the common hepatic duct (Mirizzi’s syndrome). After mobilization of the gall bladder, an intra-operative cholangiogram showed some decrease in distensibility of the bile duct, but no filling defect, tumor or stricture was identified (Figure 4). In view of the high clinical suspicion, a biopsy of the lateral wall of the bile duct wall was performed and sent for frozen section, which was reported as benign. Eventually a cholecystectomy was performed and a T-tube was placed in the bile duct for temporary external drainage of bile. Tissue from the gall bladder sent for histopathology also tested negative for malignancy. The post-operative course of our patient was uneventful, and our patient was discharged on the fifth post-operative day with normal bilirubin and hepatic enzyme levels. Our patient remains well at 18-month follow-up.

## Discussion

Mirizzi’s syndrome is a rare condition characterized by presence of a common hepatic duct obstruction caused by an extrinsic compression of an impacted stone in Hartmann’s pouch or the cystic duct. It presents in approximately 0.35% of cholecystectomies performed [4]. Predisposing factors include a long intra-mural cystic duct or a low insertion of the cystic duct into the common bile duct. Mirizzi’s syndrome was divided into two types by McSherry. In type I there is external compression on the hepatic duct without a fistula, whereas in type II a cholecystocholedochal fistula has been created by external stone compression [5]. In 1989, Csendes classified this syndrome as follows: type I, external compression and obstruction of hepatic duct; type II, cholecystobiliary fistula with erosion of less than one-third of the circumference of the hepatic duct; type III, cholecystobiliary fistula with erosion of two-thirds of the circumference; and type IV, total destruction of the hepatic duct [6]. This classification was further modified by Csendes in 2007, and type V was added; this consists of any type of the above classification plus a cholecystoenteric fistula without (type Va) or with (type Vb) gallstone ileus [7].

Patients usually present with clinical and biochemical signs of biliary obstruction, sometimes in the setting of an acute cholecystitis, acute cholangitis or pancreatitis. There is usually a chronic history of biliary symptoms and CA19-9 levels may be moderately elevated, as seen in our patient. However there have been a few case reports where very high levels of CA19-9 along with other suggestive radiological findings have been thought of as cholangiocarcinoma [3,8,9]. In our patient, the ERCP and CT scan findings were highly suggestive of neoplastic lesion involving the gall bladder and the proximal common bile duct causing luminal narrowing.

Whether CA19-9 should be used in the clinical diagnostic investigation of patients with biliary tract diseases still remains a difficult question to answer. CA19-9 is synthesized from normal human pancreatic and biliary ductal cells. Because the exact pathway between tissue and blood is unknown, the actual mechanism for elevated serum CA19-9 concentration is uncertain. It seems that extremely high and continuously increasing CA19-9 levels, together with well assessed clinical information, may point towards neoplasia [10]. However, this is not always the case as seen in our patient and also reported by others [3]. In view of such reports, the serum level of CA19-9 should probably never be regarded as a gold standard but rather as a helpful adjunct when searching for biliary malignancy. The diagnosis should always take into account medical history, clinical examination, qualitative radiology studies, and careful follow-up. If all of the above features are suggestive, a high serum CA19-9 value may be of great help in favor of a diagnosis of biliary malignancy.

Pre-operative diagnosis of Mirizzi’s syndrome is crucial in order to avoid complications of unrecognized cholecystobiliary or cholecystoenteric fistulas and damage to the common hepatic duct during surgery. For this reason, the pre-operative diagnosis requires the use of combined imaging modalities such as US, CT, ERCP, and percutaneous transhepatic cholangiography (PTC). US or CT scans are not often definitive, although both may demonstrate findings that strongly suggest a diagnosis, such as: (1) dilatation of the biliary tree above the level of the gall bladder neck, (2) impaction of a stone in the gall bladder neck, and (3) abnormal caliber common bile duct below the level of impaction. All these findings may also be present in obstruction caused by a neoplastic lesion; hence specificity remains an issue. The radiological appearance of this condition may be misinterpreted as a tumor of the gall bladder or cystic duct, a cholangiocarcinoma, metastatic disease of the hilum or acute cholecystitis [11]. A similar radiological picture raised the suspicion of cholangiocarcinoma in our patient.

ERCP is considered an effective pre-operative method for diagnosing the condition in these patients and can provide a relatively accurate localization and characterization of the cause of the biliary obstruction. Typical findings of Mirizzi’s syndrome at ERCP include (1) mid-bile duct obstruction with dilated proximal common hepatic duct and intra-hepatic ducts combined with normal duct caliber distal to the obstruction, (2) insertion of the cystic duct at the point of obstruction and/or complete obliteration of the cystic duct, and (3) a stone visualized at the point of obstruction either within the cystic duct or the common duct [4]. If a stone is not seen or suspected, however, the findings may be misleading towards a stricture or malignancy. In addition, an interesting finding that suggests Mirizzi’s syndrome indirectly during ERCP is the fact that biliary tree dilatation may subside when a patient is placed in an anti-Trendelenburg position [12].

Although diagnostic imaging techniques have been perfected, pre-operative diagnosis of Mirizzi’s syndrome is not an easy task and continues to be a challenge for the surgeon. Therefore, even intra-operative cautious recognition of the condition and application of the appropriate surgical judgment according to the characteristics of each case will lead to successful treatment. Surgical intervention remains the definitive treatment for the majority of patients and should satisfy three goals: extraction of the obstructing stone, removal of the gall bladder, and restoration of normal biliary drainage. An intra-operative cholangiogram should be obtained for clarification and confirmation [13,14]. Depending on the pre-operative diagnosis and operative findings, frozen section pathologic analysis may be indicated to assess the specimen for malignancy, as the associated incidence of carcinoma with Mirizzi’s syndrome has been noted to be as high as 27.8%, presumably secondary to chronic inflammation [15]. In our patient, the diagnosis of Mirizzi’s syndrome was made on surgical exploration and eventually a cholecystectomy with restoration of normal biliary drainage was carried out. Tissue samples sent for histopathology tested negative for malignancy.

## Conclusions

Despite improvements in clinical, biochemical and radiological investigation techniques, pre-operative diagnosis of Mirizzi’s syndrome remains a challenging task. The condition can easily be confused with choledocholilithisis, bile duct stricture or cholangiocarcinoma because of the close resemblance of radiological findings and may be overlooked due to the rarity of the condition. A high index of suspicion and careful surgical assessment may help in establishing a diagnosis and alter the clinical course for the patient. In our patient, the diagnosis of Mirizzi’s syndrome was made intra-operatively and a cholecystectomy was performed with extraction of the stone and temporary drainage of the biliary system. Our patient recovered completely with no complications within a few days.

## Consent

Written informed consent was obtained from the patient for publication of this case report and any accompanying images. A copy of the written consent is available for review by the Editor-in-Chief of this journal.

## Competing interests

The authors declare that they have no competing interests.

## Authors’ contributions

MRK conceived the study and interpreted the data. SR collected the data and prepared the initial manuscript. The manuscript was reviewed and approved by all the authors.
